# The relationship between parenting styles and cultural intelligence in undergraduate nursing students: chain mediation of humanistic caring ability and emotional intelligence

**DOI:** 10.1186/s12909-026-08847-y

**Published:** 2026-02-24

**Authors:** Yang Guo, Ruonan Wang, Anqi Li, Jie Yao, Tinghong Wang

**Affiliations:** 1 Shaanxi Institute of International Trade &Commerce, Xianyang, China; 2 University Kebangsaan Malaysia, Lumpur, Malaysia; 3 Xijing College, Xi’an, China; 4 Xi’an Peihua College, Xi’an, China; 5 Shanxi University of Traditional Chinese Medicine, Xianyang, China; 6 Department of Hematology, Gansu Provincial Hospital, Lanzhou, China

**Keywords:** Parenting styles, Humanistic caring ability, Emotional intelligence, Cultural intelligence, Undergraduate nursing students

## Abstract

**Background:**

Cultivating cultural intelligence (CQ) is crucial for preparing nursing students to provide equitable care in multicultural societies. However, the factors that contribute to CQ development in undergraduates, particularly the role of early family influences, remain underexplored. Parenting styles shape fundamental interpersonal competencies, yet their relationship with CQ, and the potential mediating roles of humanistic caring ability (HCA) and emotional intelligence(EI), have not been empirically examined.

**Objective:**

This study aimed to investigate the relationship between parenting styles (emotional warmth and rejection) and CQ in undergraduate nursing students, and to test whether HCA and EI serve as chain mediators in this relationship.

**Methods:**

This cross-sectional study was conducted on 846 undergraduate nursing students from March 2024 to September 2024 in Shaanxi Province and Gansu Province, China, using the convenience sampling. The General Information Questionnaire, Caring Competence Evaluation Scale, EI Scale, CQ Scale, and Short-form Parenting Styles Questionnaire were used to assess the undergraduate nursing students. Descriptive analysis, Pearson correlation analysis and Hayes PROCESS Macro method in SPSS 27.0 were used to test the model.

**Results:**

(1) HCA and EI significantly mediated between the parental rejection dimension and CQ, d21 paternal = 0.258, BootstrapSE = 0.152, 95%CI = (-2.054, -1.458); d21 maternal = 0.245, BootstrapSE = 0.142, 95%CI = (-2.126, -1.568). The contribution of indirect associations to the total association was 54.21%. HCA was a 25.23% mediating association, EI was a 14.86% mediating association, and the co-mediating association of HCA and EI was 14.12%. (2) HCA and EI significantly mediated the relationship between parental emotional warmth and CQ, d21 Father = 0.258, BootstrapSE = 0.123, 95%CI = (1.271, 1.756); d21 Mother = 0.240, BootstrapSE = 0.124, 95%CI = (1.453, 1.939). The contribution of indirect associations to the total association was 51.89%. HCA was a 22.88% mediating association, EI was a 17.69% mediating association, and the co-mediation association of HCA and EI was 11.38%.

**Conclusion:**

(1) These findings, derived from a sample of nursing students in Northwest China, suggest that parental rejection is negatively associated with CQ. Parental emotional warmth is positively associated with CQ. (2)HCA and EI significantly mediated this relationship. The results highlight the potential role of family factors in nursing education within similar cultural contexts. However, generalizability to other regions requires further investigation.

## Introduction

Cultural intelligence(CQ) originated abroad, in 2003, Christopher Earley and Xun Hong gave CQ and defined it as: the ability of a person (organisation) to effectively adapt to new environments, an adaptive intelligence that manifests itself in a series of adaptive, intelligent behaviours bound to the beliefs and values of new cultures and societies, and then proposed a four-dimensional structure of CQ as metacognition, cognition, motivation and behaviour [1]. Subsequently, its concept was gradually introduced into the nursing community that refers to the ability of individuals to effectively interact with others in different cultural contexts and can collect and process relevant information in different cultural situations, think differently, put themselves in the position of the other party to understand the cultural differences, make judgements and adopt effective methods of communication corresponding to them, the purpose of which is to be able to adapt to new cultural environments with the behaviour of the individual is to serve as a cultural competence core skills, plays an important role in intercultural care and is an important strategy for implementing intercultural communication [2, 3]. In actual clinical care, however, nurses need appropriate therapeutic and intercultural communication skills in order to effectively interact with different patients in different situations [4]. Therefore, the inclusion of cultural information in the study programmes of nursing students has been proposed all over the world in order to better prepare them for different populations [5]. Secondly, problematic communication in the relationship between nurses and patients leads to healthcare inequalities, patient cultural insecurity, mistrust, lack of access to healthcare, dissatisfaction with care and poor health outcomes [6]. It follows that the provision of culturally appropriate care has been recognised as integral in helping to reduce persistent and pervasive health disparities associated with healthcare access, and that in providing culturally appropriate care, healthcare professionals are able to demonstrate awareness and appreciation of different cultures and are able to tailor care to meet the individual needs of patients. In recent years, national and international studies have shown [7, 8] that nursing students must be aware of cultural diversity, and that providing care to culturally diverse patients to meet their healthcare needs may lead to patient isolation and dissatisfaction, and that CQ has been identified as an important factor in improving the cultural competitiveness of undergraduate nursing students [9]. In addition to this, improving the CQ of undergraduate nursing students can reduce conflict due to cultural differences, facilitate communication between individuals from different cultural backgrounds, and is an indispensable factor that directly influences whether or not nursing students use an integrative conflict management model when dealing with conflicts with patients [10].

## Background

### Importance of parenting styles

According to Bronfenbrenner’s ecosystem theory, the family, as a micro-system, has the most direct influence on the individual [11], and family education is the earliest, longest, and most basic education received by students, so the family education field plays a direct role in the level of students’ individual literacy. Parenting style as a stable parenting style and behavioural tendency of parents in the process of raising children, is a synthesis of parenting attitudes, concepts and behaviours, reflecting the nature of parent-child relationship, and its parenting style plays an important role in the growth of an individual, and plays an important role in the cultivation of a sound personality and positive and optimistic character of an individual [12, 13]. While recognizing the increasing diversity of family structures (e.g., single-parent, LGBTI+ families) in contemporary society, the parent-child relationship remains one of the most fundamental and enduring influences on an individual’s early development [14]. This study focuses on perceived parenting styles as a key aspect of this relationship, acknowledging that its manifestations and impacts may vary across different family contexts, an important area for future research.

Research has shown [15] that parenting styles can have a direct impact on children’s development through targeted parenting styles, as well as a subtle impact on children’s development through non-targeted parenting styles, and play a decisive role in family education. Another study pointed out [16] that parenting style plays a certain moderating role in the process of internalising humanistic literacy in the cognitive strengths possessed by college students. Specifically, college students with moderate parenting styles have cognitive strengths that dominate the formation of humanistic literacy, and their levels of judgment and insight are stronger. Humanistic literacy is the humanistic heritage, which contains three factors: humanistic accumulation, humanistic sentiment and aesthetic interest. Families with kind upbringing can give their children more positive emotions, and children who grow up in love and care are more likely to respect others, and their ability to love learning is relatively high, which makes their children’s desire for beautiful things stronger, sympathetic, and their character more delicate, sentimental and imaginative. Therefore, the parenting style plays a key role in the formation of cultural education and humanistic qualities in universities.

### The potential mediating role of HCA and EI

First of all, nursing Humanistic caring ability (HCA) refers to the ability of nursing workers to externalise their own internal humanistic qualities to serve patients autonomously [17], which refers to understanding the cultural background of patients, respecting the values of patients, expressing the caring emotions of nursing staff, coordinating the interpersonal relationships of patients, and implementing personalised care according to the needs of patients [18]. Nursing students are the vital force and important reserve force of clinical nursing, and the level of nursing students’ HCA is crucial to the improvement of clinical nursing service level [19]. It was found [20] that family education has a subtle influence on the HCA of nursing students, and its positive influence can help nursing students develop an independent character, a responsible attitude towards patients and a helpful mentality, so that nursing students have more courage and confidence in the process of caring for and taking care of patients. Specifically, parents give nursing students more warmth and care through gentle emotional education, which can increase their subjective sense of well-being, improve their motivation to work in the clinic, and thus promote the development of their HCA [21]. When nursing students encounter negative parenting styles, they are prone to form a humble, introverted personality, poor communication with others, lack of pro-social competence, and lack of the courage to take the initiative to care for others [22], and at the same time, produce anxiety and depression, while this adverse emotion is directly associated with the quality of their work and the physical and mental health of the patients, so that the nursing student’s motivation to work decreases, thus impeding the enhancement of their caring ability.

Emotional intelligence (EI) refers to the ability to assess changes in one’s own and others’ emotions, to recognize different emotions, and to use this information to guide one’s thinking and actions [23]. EI has a subtle effect on individuals, and the level of EI has a facilitating or inhibiting effect on the success of individuals. EI is EI, which is a better predictor of an individual’s success than IQ, and is defined as a number of emotional and social abilities that facilitate an individual’s ability to cope with the demands of daily life. Chinese scholar Zhang Qingzhi’s study showed [24] that all four dimensions of college students’ EI were significantly correlated with parenting styles, and that healthy parenting styles would have a positive effect on the development of an individual’s EI, whereas vice versa, they would have a negative effect and hinder the development of an individual’s EI.

In addition, there is a significant correlation between HCA and EI [25], and nursing interns with high EI can maintain a keen and detailed awareness in the process of clinical internship and different hospital environments, so that they can integrate themselves into their classmates’ groups, patients’ family members’ minds and even the society more quickly, and embody a sense of caring. In addition, individuals with a high level of EI have a strong ability to manage their own emotions, and when encountering problems, they can put themselves in the position of others to think about the problem, and can give care and help to others when solving the problem [26]. Another study pointed out [27], undergraduate nursing students’ HCA is significantly positively correlated with CQ, and its findings show that nursing students’ family background, cultural background, educational concepts and methods have a greater degree of specificity, which leads to their inability to fully understand the differences between the cultures of different groups of people, and bias in the understanding of humanistic caring methods, which increases the risk of conflict in interactions. In addition, it was also pointed out [26] that nursing students with high CQ have a keen and meticulous awareness, and can implement a caring approach in accordance with different hospital environments and the cultural needs of patients, so as to meet the cultural needs of patients while implementing high-quality humanistic care services. In addition, Dramed et al. showed that [28], the EI of college students is significantly positively correlated with CQ, and nursing students with high EI can better regulate their suppressed emotions, learn how to serve negative emotions, think logically in different environments, realize the importance of multicultural care in clinical work, and develop their own cross-cultural communication and cultural recognition skills, thus improving their CQ level. They also realize the importance of multicultural nursing in clinical work, and develop their intercultural communication and cultural recognition skills, thus improving their CQ.

### From the family microsystem to the development of CQ

This study is anchored in two complementary theoretical perspectives. First, Bronfenbrenner’s ecological systems theory posits that the family, as the most immediate microsystem, exerts a direct and powerful influence on individual development [11]. Within this microsystem, parenting styles constitute a primary mechanism through which the family environment shapes a child’s competencies and worldviews. Second, the “parent-child transfer” theory provides a mechanism for this influence, suggesting that parents’ behaviors, emotions, and cognitions are implicitly transmitted to their children, fostering the development of similar patterns [29]. Therefore, we propose a sequential pathway to understand the development of CQ in nursing students. We posit that parenting styles (the microsystem mechanism) lay the foundation for two key interpersonal competencies: HCA (an outward-oriented, prosocial competency) and EI (an inward-oriented, self-regulatory competency). These competencies, cultivated through familial “transfer”, are theorized to serve as essential personal resources. In turn, they equip individuals to more effectively navigate, understand, and adapt to diverse cultural contexts, thereby enhancing CQ.

### Aims

**Fig. 1:**
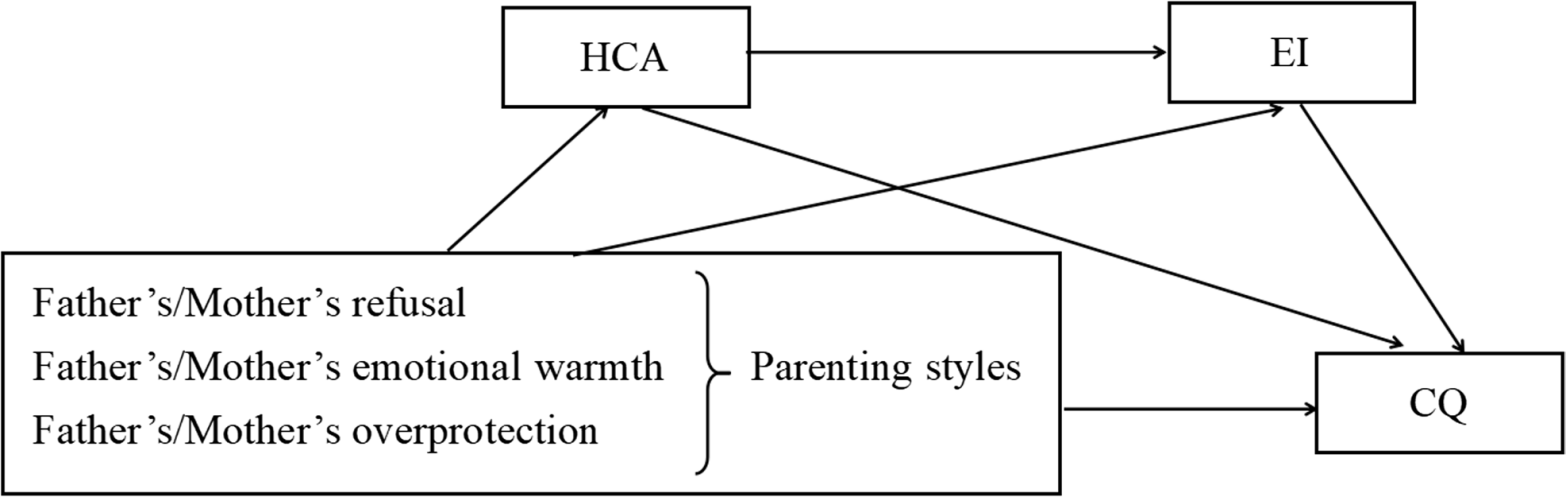
Theoretical framework of this study

At present, scholars at home and abroad pay more and more attention to the level of CQ of corporate employees and international students, but there are fewer studies in the field of nursing, and little attention is paid to the CQ of nursing students, but clinical nursing work is closely related to cultural nursing. After reviewing the literature, this study introduced the “parent-child migration” theory to develop a mediation model, and by using the parenting style of parents as the independent variable, the level of CQ as the dependent variable, and HCA and EI as the mediator variables, the HCA and EI in the relationship between parenting styles and CQ are investigated. The chain mediating role between parenting styles and CQ was studied and analysed, and hypotheses were made (Fig. 1): Hypothesis 1 (H1): Parental emotional warmth will be positively correlated with CQ, whereas parental rejection will be negatively correlated with CQ. Hypothesis 2 (H2): Parenting styles (emotional warmth and rejection) will show a direct association with the CQ level of undergraduate nursing students. Hypothesis 3 (H3): HCA will mediate the relationship between parenting styles (emotional warmth and rejection) and CQ. Hypothesis 4 (H4): EI will mediate the relationship between parenting styles (emotional warmth and rejection) and CQ. Hypothesis 5 (H5): HCA and EI will sequentially mediate (chain mediate) the relationship between parenting styles (emotional warmth and rejection) and CQ.

## Methods

### Design

A cross-sectional survey was conducted from March 2024 to September 2024.

### Participants

Selection of research subjects: undergraduate nursing students enrolled in region of Shaanxi Province and Gansu Province. (1) Inclusion criteria:① undergraduate nursing students enrolled in universities in district, Shaanxi and Gansu Province. ② Students with a clear sense of autonomy and informed consent who voluntarily participated in the survey. (2) Exclusion criteria: ① Students who are on leave of absence, changing majors, or about to drop out of school. ② Students who self-reported a diagnosed psychological disorder (e.g., depression, bipolar disorder) or were under current psychiatric treatment. A priori power analysis was conducted using GPower 3.1.9.7 [30]. To detect a small to medium effect size (f²= 0.08) in a multiple linear regression model with up to 10 predictors, with an alpha level of 0.05 and a desired power of 0.80, the minimum required sample size was 118. Considering potential invalid responses and to ensure robust statistical power for the more complex mediation analysis, we aimed to recruit a substantially larger sample. A total of 846 participants were ultimately enrolled, far exceeding the minimum requirement.

### Instruments

The selection of measurement instruments was directly informed by our theoretical framework. To assess the key characteristic of the family microsystem, we employed the Short-form Parenting Style Questionnaire (SPSQ), which measures the child’s perception of parental behavior, which a core construct in ecological theory emphasizing the subjective experience of the environment. To measure the personal competencies theorized to develop within this microsystem, we used the Caring Competence Evaluation Scale (for HCA) and the EI Scale. Finally, to assess the adaptive outcome relevant to broader systems, we used the CQ Scale. All instruments are self-report, aligning with the theoretical focus on the individual’s phenomenological experience within their ecological context.

(1) General Information Questionnaire: a self-developed general information questionnaire, including 13 entries such as whether the parents of undergraduate nursing students are in employment, whether their economic status is, good, the type of family education, their relationship with their parents, and their willingness to work in nursing after graduation.

(2) Caring Competence Assessment Scale (CCAS): Developed by Nkongho (1990) [31], this 37-item scale measures HCA across three dimensions: patience (13 items), courage (13 items), and caring (11 items). Items are rated on a 7-point Likert scale (1 = strongly disagree to 7 = strongly agree), with 13 items reverse-scored. The total score ranges from 37 to 259, with higher scores indicating greater caring competence. In this study, the Cronbach’s ɑ for the total scale was 0.911. CFA supported the original three-factor structure, demonstrating acceptable model fit: χ²/df = 2.31, CFI = 0.924, TLI = 0.915, RMSEA = 0.049. All factor loadings were significant (*p* < 0.001), ranging from 0.52 to 0.86.

(3) EI Scale: The scale compiled by Schutle et al. based on Mayer and Salovey’s model and translated into Chinese by Wang Caikang [32] was used. It contains 33 items across four dimensions: emotional perception (10 items), self-emotion regulation (9 items), regulation of others’ emotions (8 items), and use of emotions (6 items). Responses are recorded on a 5-point Likert scale (1 = very unconformable to 5 = very conformable). The theoretical median is 99. In this study, the overall Cronbach’s ɑ was 0.892. CFA for the four-factor model yielded satisfactory fit indices: χ²/df = 3.05, CFI = 0.908, TLI = 0.901, RMSEA = 0.055. Standardized factor loadings ranged from 0.55 to 0.82.

(4) The CQ Scale (CIS): The scale developed by Ang et al. [33] and translated by Wang Qiqi et al. [34] assesses CQ through 20 items in four dimensions: metacognition (4 items), cognition (6 items), motivation (5 items), and behavior (5 items). A 7-point Likert scale (1 = completely incompatible to 7 = completely compatible) is used, with total scores from 20 to 140. In this study, Cronbach’s ɑ was 0.920. CFA confirmed the hypothesized four-factor structure: χ²/df = 2.89, CFI = 0.938, TLI = 0.927, RMSEA = 0.053. Furthermore, discriminant validity was established as the square root of the Average Variance Extracted (AVE) for each factor (ranging from 0.71 to 0.79) was greater than its correlations with other factors.

(5) The Simplified Parenting Style Questionnaire: The Chinese version revised by Jiang et al. [35] was employed. It comprises two identical 21-item sections for father and mother, each containing three dimensions: rejection (6 items), emotional warmth (7 items), and overprotection (8 items). A 4-point Likert scale (1 = never to 4 = always) is used. In this study, Cronbach’s ɑ for the total scale was 0.80 for both father and mother forms. Separate CFAs for father and mother models supported the three-factor structure. For the father form: χ²/df = 3.42, CFI = 0.916, TLI = 0.905, RMSEA = 0.061. For the mother form: χ²/df = 3.28, CFI = 0.919, TLI = 0.909, RMSEA = 0.059. All items loaded significantly on their respective factors (loadings: 0.58–0.85).

### Data collection

#### Stage 1: Establish a research group and conduct a literature review

- (1)Set up a research team, consisting of four faculty member and three graduate students, using Wipro, Wanfang, pubmed and other databases, undergraduate nursing students, medical students, nursing majors, parenting styles, parenting education, family education, HCA, EI, CQ, cultural nursing and other keywords, to review and understand the information of relevant studies at home and abroad.
- (2) After reviewing the information, the group members determined the purpose, content, and object of the study, selected the research tools, and formulated the questionnaire.

#### Stage 2: Distribution and recovery of questionnaires

- (1)Pre-survey stage: 200 undergraduate nursing students of Shaanxi Insititute of International Trade & Commerce are selected for pre-survey by convenience sampling method; through pre-survey, we find out the problems of the self-drafted general information questionnaire, and delete or repair unreasonable entries. We record the problems that occur during the process of distributing and collecting questionnaires, sum up the experience, and make modifications to the survey programme in order to ensure the smooth progress of the formal survey.
- (2)Formal survey: according to the purpose of the study, this study used a convenience sampling method, selecting undergraduate nursing students from 3 medical colleges and universities in Guanzhong area of Shaanxi Province, and 2 medical colleges in Gansu Province. It should be noted that convenience sampling may introduce selection bias and limit the generalizability of the findings to other populations. The screening the respondents based on the nativity criteria. Before distributing the questionnaire, contacting the nursing professional in charge of each university, the questionnaire used in the study was sent for review, and after passing the review, the questionnaire was distributed by choosing the way of online distribution, and it was distributed and recovered by adopting the questionnaire star with the consent of the respondents in an anonymous filling manner.The questionnaires were collected from March 2024 to September 2024. The study was reviewed and approved by the School of Medicine, Shaanxi Institute of International Trade & Commerce Ethics Committee(Ethical approval number: HLX20240101332), and was conducted in accordance with the Declaration of Helsinki.

#### Stage 3: Analysing the data

There were 870 questionnaires initially collected, 24 were excluded due to extensive missing data (>20% of items) or patterned responses. For the remaining 846 valid questionnaires, missing values were minimal (<2% for any variable) and were handled using expectation-maximization (EM) imputation. The collected data were analysed statistically using SPSS27.0 software, and the results were compared with the existing research findings in the literature review, summarised and concluded in this study.

### Statistical analysis

The collected data were collated and numbered sequentially, the database of this study was created using an Excel sheet with two-person entry, and the error-free data were statistically analysed using SPSS27.0 with a significance level of α = 0.05, and considered statistically significant at *P* < 0.05. Counts were described statistically using mean±standard deviation, frequency and composition ratio. Pearson correlation was used to analyse the relationship between parenting styles, humanistic caring competence, EI and CQ among undergraduate nursing students. Process plug-in was used to construct structural equations, and humanistic caring competence and EI were used as mediator variables for the mediation effect analysis.

### Ethical considerations

This study strictly adhered to fundamental ethical principles throughout the research process. First, the principle of voluntary participation was upheld, ensuring that all participating university students and group members retained the right to withdraw from the study at any time without providing a reason. Second, the principle of informed consent was followed by fully informing all respondents of the survey’s purpose, significance, and procedures prior to participation, after which they provided electronic informed consent. Finally, the principle of confidentiality was maintained by ensuring all collected information was anonymized and securely handled, with no personally identifiable data disclosed in any part of the study.

### Validity and reliability/rigour

All instruments used in the study were adapted and validated for the Chinese culture and had good validity and reliability. In addition, all investigators were trained in registration, checking the completeness of the questionnaires, and the ethical principles of conducting research prior to the formal survey. To reduce the risk of self-reporting bias, the information of all present investigators was kept strictly confidential. Finally, to ensure the rigour and accuracy of the statistical analysis, a statistician was invited to check the data processing.

## Results

### Common method bias test

The Harman’s single-factor test was conducted to assess the threat of common method bias. All items from the key scales were subjected to an exploratory factor analysis with unrotated principal component factoring. The results extracted 23 factors with eigenvalues greater than one. The first factor accounted for 23.648% of the total variance, which is below the commonly recommended threshold of 40% or 50% [28]. While this suggests that common method bias is unlikely to be a severe threat to the validity of our findings, it is important to note that Harman’s single-factor test is a diagnostic technique with limitations. It may not detect more complex forms of common method variance, and its diagnostic power has been debated in methodological literature [36].

### Demographic information about the participants

The vast majority of the respondents were female students, accounting for 84.5 per cent, while more than 75 per cent of the nursing students were older than 18 years of age, and the proportion of Han Chinese students was as high as 90%, and more students who participated in the survey were freshmen. In this survey, more than 75% of the students come from rural areas, about 20% of the students are class cadres, less than 20% of the students are only children, less than 10% of the students’ parents are working, more than 50% of the students’ family economic situation needs to be further improved, 70% of the parents’ family education is democratic, and nearly 90% of the students have a better relationship with their parents, and more than 80% of the students would like to continue to work in nursing after graduation.

### Status of parenting style, HCA, EI and CQ

As shown in Table 1, the scores of 846 nursing students on the dimensions of parenting styles were, in descending order, overprotection, emotional warmth, and rejection. It can be seen that in family education, children feel more overprotection from their parents, followed by emotional warmth from their parents, and parents are less likely to use negative and harsh parenting styles for their children. The total score of HCA of nursing students is (175.20 ± 34.59), which is in the middle level according to the scoring criteria. The total score of EI is (120.49 ± 20.00), which is greater than the theoretical median value of 99, so the EI of nursing students is higher. The total score of CQ is (91.67 ± 17.37), which is in the middle level according to the scoring criteria.

**Table 1:** Parenting style, HCA, EI and CQ scores (X ± S, points)

varible	maximum	minimum	totals	Average question score
The Father refuse	23.00	6.00	12.60 ± 3.66	2.10 ± 0.61
Father’s emotional warmth	28.00	7.00	15.32 ± 4.47	2.18 ± 0.64
Father’s overprotective	31.00	8.00	16.56 ± 4.78	2.07 ± 0.60
The mother refuse	23.00	6.00	13.03 ± 3.85	2.17 ± 0.64
Mother’s emotional warmth	27.00	7.00	15.77 ± 4.38	2.25 ± 0.63
Mother’s overprotective	31.00	8.00	17.00 ± 4.97	2.13 ± 0.62
HCA	257.00	74.00	175.20 ± 34.59	4.73 ± 0.93
patience	98.00	18.00	63.07 ± 15.19	6.31 ± 1.52
bravery	91.00	22.00	65.07 ± 13.68	5.00 ± 1.05
cognitive	70.00	13.00	47.06 ± 10.47	3.36 ± 0.75
EI	164.00	63.00	120.49 ± 20.00	3.65 ± 0.61
emotional awareness	60.00	18.00	42.09 ± 8.49	3.51 ± 0.71
self-emotion control	40.00	10.00	29.33 ± 5.68	3.67 ± 0.71
emotional regulation of others	30.00	9.00	22.30 ± 4.37	3.71 ± 0.73
use of Emotions	35.00	12.00	26.77 ± 4.84	3.82 ± 0.69
CQ	138.00	33.00	91.67 ± 17.37	4.58 ± 0.87
metacognition	28.00	6.00	19.32 ± 4.11	4.83 ± 1.03
cognitive	42.00	7.00	26.34 ± 6.29	4.39 ± 1.05
motivations	35.00	8.00	22.68 ± 5.20	4.54 ± 1.04
action	35.00	10.00	23.33 ± 4.87	4.67 ± 0.97

### Correlation between parenting style, HCA, EI and CQ

From the results of Table 2, it can be seen that parental rejection dimension is significantly and negatively correlated with HCA, EI, and CQ (*r*=-0.304, *r*=-0.335, and *r*=-0.371), (*r*=-0.357, *r*=-0.366, and *r*=-0.410). Parental emotional warmth dimension is significantly and positively correlated with HCA, EI, and CQ (*r* = 0.309, *r* = 0.395, *r* = 0.388), (*r* = 0.379, *r* = 0.357, *r* = 0.427), therefore, Hypothesis 1 has been tested. And there was no correlation between parental overprotection and any of the variables.

**Table 2:** Correlation between parenting style, HCA, EI and CQ (r)

varible	1	2	3	4	5	6	7	8	9
1	1								
2	0.213^*^	1							
3	-0.196^*^	-0.147^*^	1						
4	-0.204^*^	-0.251^*^	0.271^*^	1					
5	0.070^#^	/	/	/	1				
6	/	/	/	/	0.252^*^	1			
7	-0.304^*^	-0.357^*^	0.309^*^	0.379^*^	/	/	1		
8	-0.335^*^	-0.366^*^	0.395^*^	0.357^*^	/	/	0.497^*^	1	
9	-0.371^*^	-0.410^*^	0.388^*^	0.427^*^	/	/	0.540^*^	0.510^*^	1

1 to 9 represent: father’s refusal, mother’s refusal, father’s emotional warmth, mother’s emotional warmth, father’s overprotection, mother’s overprotection, HCA, EI, and CQ, respectively

^#^*P* < 0.05

^*^P < 0.01

### Mediating effects of HCA and EI

- (1)Analysis of the mediating effect of the parental rejection dimension.

The chain-mediated effects of HCA and EI were analyzed using the PROCESS Macro Model 6. After controlling for age, gender, family economic status, and self-reported prior cross-cultural experience, the parental rejection dimension was found to be directly and negatively associated with nursing students’ CQ (c father = -1.756, t = -11.559, p < 0.001. c mother = -1.847, t = -13.010, p < 0.001). When HCA and EI were included in the regression equation simultaneously, the parental rejection dimension remained a significant negative predictor of CQ (c’ father = -0.805, t = -5.864, *p* < 0.001. c’mother = -0.851, t = -6.406, *p* < 0.001), supporting Hypothesis 2.

Further analysis showed that the parental rejection dimension was significantly and negatively associated with HCA (a1 father = -3.164, t = -10.308, *p* < 0.001. a1 mother = -3.285, t = -11.389, *p* < 0.001), which in turn was positively associated with CQ (b1 father = 0.140, t = 8.782, *p* < 0.001. b1 mother = 0.137, t = 8.610, *p* < 0.001). Similarly, parental rejection dimension was a significant negative associated with EI (a2 father = -0.858, t = -5.017, *p* < 0.001. a2 mother = -1.105, t = -6.470, *p* < 0.001), and EI was positively associated with CQ (b2 father = 0.304, t = 11.150, *p* < 0.001. b2 mother = 0.293, t = 10.684, *p* < 0.001). Shown as Table 3.

**Fig. 2:**
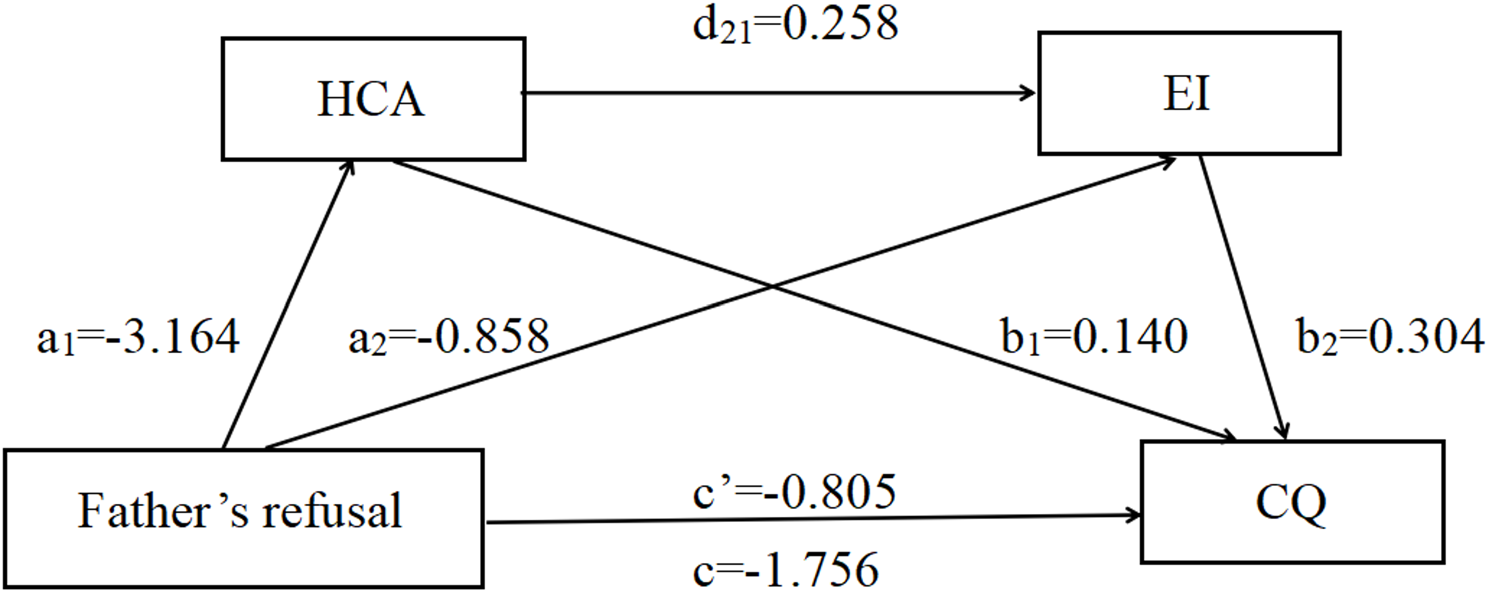
Results of mediation associations of father rejection dimension

**Fig. 3:**
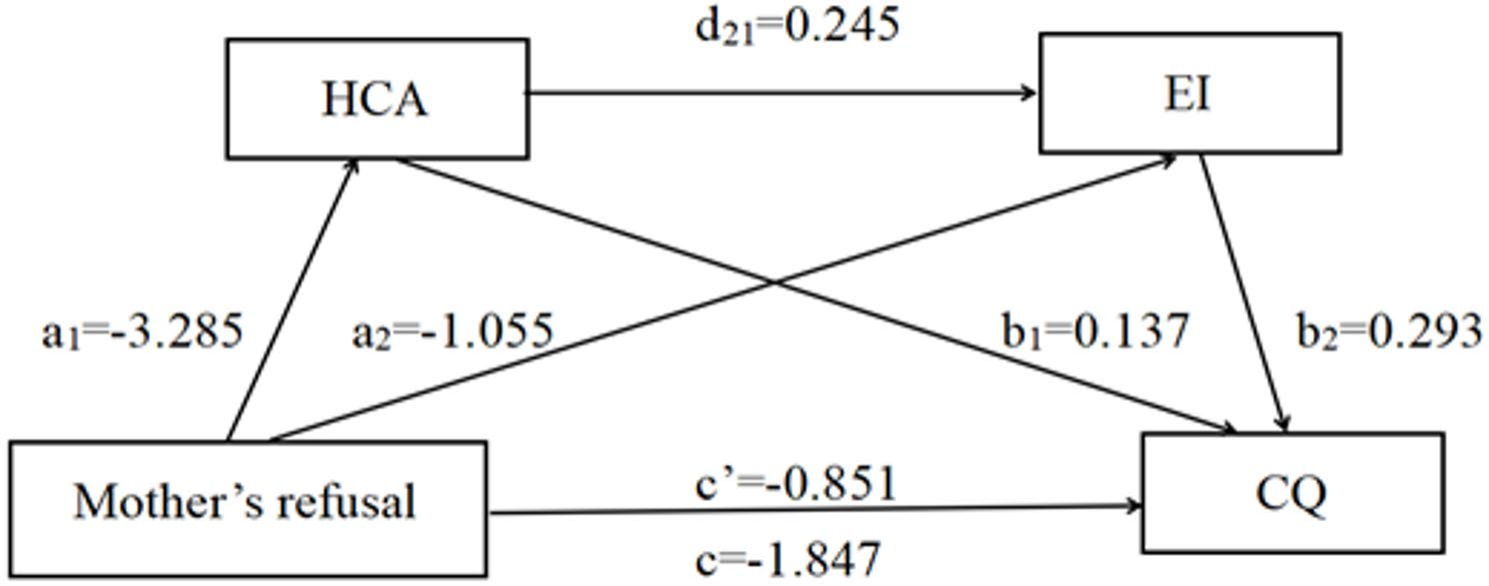
Results of mediation associations of mother rejection dimension

Bias-corrected percentile Bootstrap tests indicated that both HCA and EI significantly mediated the relationship between parental rejection (both paternal and maternal) and CQ. The indirect effects were as follows: for the paternal model, effect = -0.258, Bootstrap SE = 0.152, 95%CI (-2.054, -1.458). For the maternal model, effect = -0.245, Bootstrap SE = 0.142, 95%CI (-2.126, -1.568). The total indirect association accounted for 54.21% of the total association. Specifically, the mediating association of HCA accounted for 25.23% (supporting Hypothesis 3), EI accounted for 14.86% (supporting Hypothesis 4), and their chain mediation accounted for 14.12% (supporting Hypothesis 5). The direct, indirect, and total associations are illustrated in Figs. 2 and 3.

**Table 3:** Mediating association of HCA and EI on the parental rejection dimension

Father’s refuses	effect	Boot SE	Boot LLCI	Boot ULCI	Ratio of indirect to total effect
Total association	-1.756	0.152	-2.054	-1.458	-
Direct association	-0.805	0.137	-1.074	-0.535	-
Total indirect association	-0.952	0.095	-1.133	-0.771	54.21%
Ind1	-0.443	0.068	-0.586	-0.320	25.23%
Ind2	-0.261	0.057	-0.372	-0.153	14.86%
Ind3	-0.248	0.037	-0.327	-0.180	14.12%
C1	-0.182	0.095	-0.375	0.001	-
C2	-0.195	0.070	-0.337	-0.060	-
C3	-0.013	0.068	-0.142	0.124	-
Mather’s refuses					
Total association	-1.847	0.142	-2.126	-1.568	-
Direct association	-0.851	0.132	-1.112	-0.590	-
Total indirect association	-0.996	0.096	-1.189	-0.811	53.93%
Ind1	-0.450	0.067	-0.588	-0.325	24.36%
Ind2	-0.309	0.057	-0.428	-0.203	16.73%
Ind3	-0.236	0.035	-0.309	-0.173	12.78%
C1	-0.141	0.095	-0.326	0.046	-
C2	-0.214	0.070	-0.355	-0.082	-
C3	-0.073	0.060	-0.197	0.042	-

- (2)Analysis of the mediating effect of parental emotional warmth dimension.

To test Hypotheses 2–5 concerning mediation, Hayes’ PROCESS Macro Model 6 was employed. The analysis first examined the model with parental rejection dimension as the independent variable. After controlling for age, gender, family economic status, and prior cultural exposure, the total, direct, and indirect association are summarized in Table 4. Key findings were as follows: the parental emotional warmth dimension was directly associated with CQ (c’ father = 1.513, t = 12.264, *p* < 0.001. c’ mother = 1.696, t = 13.717, *p* < 0.001), and when HCA and EI were entered into the regression equation at the same time, the association of the parental emotional warmth dimension on CQ remained significant (c’ father = 0.681, t = 5.937, *p* < 0.001. c’ mother = 0.816, t = 6.983, *p* < 0.001). Hypothesis 2 was tested.

The parental emotional warmth dimension was a significant positively associated with HCA (a1 father = 3.065, t = 12.510, *p* < 0.001. a1 mother = 2.824, t = 11.099, *p* < 0.001), and HCA was a significant associated with of CQ (b1 humanistic (father) = 0.132, t = 8.138, *p* < 0.001. b1 humanistic (mother) = 0.138, t = 8.721, *p* < 0.001). The parental emotional warmth dimension was a significant positive associated with EI (a2 father = 0.591, t = 4.096, *p* < 0.001. a1 mother = 1.056, t = 7.451, *p* < 0.001). EI was a significant positively associated with CQ ((b2 emotional (father) = 0.309, t = 11.384, *p* < 0.001). b2 emotional (maternal) = 0.284, t = 10.302, *p* < 0.001)).

**Fig. 4:**
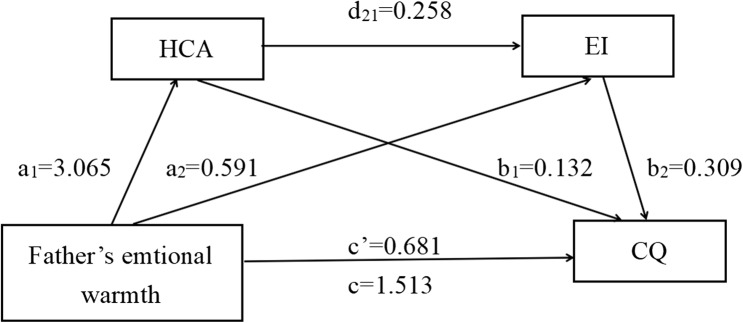
Results of mediation associations of father emotional warmth dimension

**Fig. 5:**
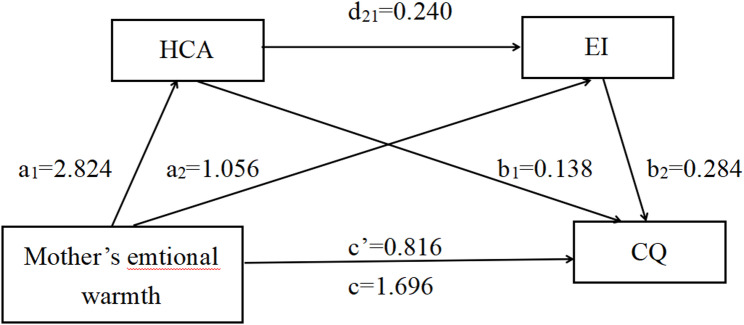
Results of mediation associations of mother emotional warmth dimension

Percentile bias-corrected Bootstrap method tests indicated that HCA and EI significantly mediated the relationship between paternal/maternal emotional warmth and CQ, d21 paternal = 0.258, BootstrapSE = 0.123, 95%CI=(1.271, 1.756). d21 maternal = 0.240, BootstrapSE = 0.124, 95%CI =(1.453, 1.939). The contribution of indirect association to the total association was 51.89%. HCA was 22.88% of the mediating association (supporting Hypothesis 3). EI was 17.69% of the mediating association(supporting Hypothesis 4). The co-mediating association of HCA and EI was 11.38% (supporting Hypothesis 5). Figs. 4 and 5 show the direct, indirect, and total associations.

**Table 4:** Mediating associations of HCA and EI on Parental Emotional Warmth

Father’s emtional warmth	effect	Boot SE	Boot LLCI	Boot ULCI	Ratio of indirect to total effect
Total association	1.513	0.123	1.271	1.756	-
Direct association	0.681	0.115	0.456	0.906	-
Total indirect association	0.832	0.082	0.669	0.995	54.99%
Ind1	0.405	0.061	0.292	0.532	26.77%
Ind2	0.183	0.047	0.092	0.275	12.10%
Ind3	0.244	0.034	0.184	0.313	16.13%
C1	0.223	0.080	0.073	0.384	-
C2	0.161	0.068	0.028	0.296	-
C3	-0.062	0.059	-0.186	0.047	-
Mather’s emtional warmth					
Total association	1.696	0.124	1.453	1.939	-
Direct association	0.816	0.117	0.586	1.045	-
Total indirect association	0.880	0.080	0.730	1.039	51.89%
Ind1	0.388	0.058	0.278	0.507	22.88%
Ind2	0.300	0.049	0.208	0.401	17.69%
Ind3	0.193	0.028	0.141	0.252	11.38%
C1	0.089	0.084	-0.075	0.252	-
C2	0.196	0.061	0.077	0.314	-
C3	0.107	0.051	0.005	0.209	-

## Discussion

### Theoretical interpretation of key findings

Guided by Bronfenbrenner’s ecological systems theory which posits the family as a foundational microsystem, and the “parent-child transfer” hypothesis which explains the transmission of behavioral patterns, this study investigated a sequential pathway from parenting to CQ. Our results offer strong empirical support for this integrated framework. The significant associations we observed that where in parental emotional warmth positively, and rejection negatively, correlated with HCA, EI, and ultimately CQ, directly illustrate the profound and enduring impact of the family microsystem. Crucially, the validated chain mediation model elucidates the mechanism of this impact: parenting styles appear to first cultivate (or hinder) key personal competencies (humanistic caring and EI), which in turn serve as the proximal resources enabling effective adaptation in multicultural settings. This sequential “transfer” of influence from the family environment to internal competencies, and then to external adaptive capability, provides a nuanced explanation consistent with our guiding theories. The following sections delve into the specifics of these associations.

### Status of nursing students’ parenting styles, HCA, EI and CQ

The results of this study showed that the scores of the dimensions of parenting styles of undergraduate nursing students in Guanzhong region were emotional warmth, rejection, and overprotection in descending order, which indicated that parents tended to create a warm family environment for their children to educate them, which was consistent with the results of previous studies [20]. The reason for this analysis may be that Chinese-style family education tends to pamper children more, and parents give more care to their children to make them feel the warmth of the family in order to facilitate the growth and development of their children. The total score of HCA is (175.20 ± 34.59), which can be seen from the scoring criteria that the HCA of nursing students is at a medium level, and the scores of the dimensions of HCA were patience, courage, and cognition in descending order, which is consistent with the findings of other scholars [19–21]. Analyse the reasons may be the following two points: First, the majority of the survey respondents belong to the non-only child, they grew up with their siblings, many students act as elder brother, elder sister, know how to take care of others and think about others, and the sense of caring for and taking care of others is stronger. Secondly, the majority of the target of this survey are undergraduate nursing students in school, and although they have learnt the theoretical knowledge of humanistic care during their school years, they lack the experience of clinical practice, the level of cognition of things is not enough, and they lack the courage and ability to deal with unknown things.

Therefore, the nursing students’ cognitive level of HCA is low, and the courage and ability to take the initiative to care for others and deal with unknown things are insufficient. The total score of EI is (120.49 ± 20.00), which is higher than the theoretical median of 99, therefore, the EI is at a high level, which indicates that the nursing students have a better ability to regulate their emotions, which is explained by the fact that. On the one hand, the vast majority of the respondents of this study are females, and females are more gentle and understanding in their character, and they have a higher level of sympathy and empathy than males, and they are good at understanding their own emotions and the emotions of others, and they know how to manage these emotions effectively [37].On the other hand, the essence of the nursing profession is to care, care, and establish a good social relationship with patients in clinical work, so how to effectively carry out emotional regulation is particularly important. Emotional perception needs to be obtained through more communication, and more than 80% of the respondents of this survey are school nursing students, whose emotional response is in the period of instability, and lack of mastery of effective regulation of their own emotions, and nursing students have not yet entered the clinical work, and their interpersonal communication ability is insufficient, which leads to a lower score of their emotional perception dimension.

The total score of CQ was (91.67 ± 17.37), and according to the scoring criteria, it is known that the CQ of nursing students is at a medium level, which is consistent with the results of other studies [10]. Among the four dimensions of the CQ scale, the metacognitive dimension scored the highest, probably due to the fact that nursing undergraduates were able to realise that both parties were in different cultural environments when interacting with people and consciously changed the way they dealt with problems, reflecting on the problems and checking for flaws in their communication. The cognitive dimension scored the lowest, probably due to the fact that nursing undergraduates were located in a relatively homogeneous campus environment, with fewer chances to confront people from different cultural backgrounds and identify the cultural backgrounds of different individuals, and thus were not able to adopt effective communication skills to cope with them.

### Parenting with emotional warmth contributes to the development of HCA, EI and CQ

The results of the study showed that the dimension of parental emotional warmth was significantly and positively associated with nursing students’ humanistic competence, EI and CQ (*P* < 0.01). First, this study draws upon the “parent-child transfer” mechanism within Bronfenbrenner’s microsystem to examine the influence of an emotionally warm parenting style. Characterized by tolerance, understanding, and respect towards children’s daily behaviors, this parenting approach provides a stable and secure emotional base, thereby facilitating the intergenerational transfer of prosocial attitudes and emotion regulation skills. Previous studies are largely consistent with the findings of this study. When parents adopt an emotionally warm approach, their children are more likely to develop kind and gentle personality traits, exhibit enhanced prosocial abilities, and demonstrate greater awareness and patience in caring for others [20]. Tian Guixiang et al. [38, 39] found that parents who give nursing students more warmth and care through emotional warmth parenting can increase the subjective well-being of nursing students, improve their motivation to work in the clinic, and thus improve the HCA of nursing students. Secondly, parenting styles are significantly correlated with college students’ emotional expressiveness, with positive approaches such as emotional warmth facilitating the development of EI. Specifically, when parents adopt an emotionally warm style, children are less likely to use expressive suppression strategies in daily life. This supportive environment encourages emotional expression, fulfills emotional needs, and provides a foundation for the healthy growth of EI. This study further identified a significant positive correlation between parental emotional warmth and CQ. We posit that parents who establish positive parent-child relationships and cultivate a warm, harmonious family atmosphere implicitly shape their children’s values and educational outlook. Such a nurturing environment contributes to the development of children’s cultural literacy, guiding them to adapt to diverse cultural settings, respect cultural differences, and appreciate aesthetic diversity. As a result, children tend to exhibit greater empathy, a more nuanced character, and a stronger inclination toward understanding and engaging with multicultural contexts.

### Parental rejection dimension hinders the development of HCA, EI and CQ

The results of this study showed that the parental rejection dimension was significantly negatively correlated with nursing students’ HCA, EI and CQ (*P* < 0.01). First of all, parenting style of parental rejection refers to parents giving punishments, chastisement, etc. to their children when they make mistakes or being too strict in disciplining and supervising their children’s daily behaviours. Previous studies have shown that [22], parents use the rejection style to treat their children, the children are prone to be introverted and withdrawn, like to be alone, the ability to communicate and exchange with others is reduced, and the lack of active caring for others consciousness. Nursing students who are in a negative family education environment for a long time will produce negative emotions such as nervousness, distrust, etc., which directly associated with their performance in clinical work, and they are prone to errors and burnout, which decreases their motivation and the quality of nursing services, thus hindering the development of their caring ability. Secondly, according to the theory of “parent-child migration” [29], parents, as the first significant others contacted by their children, will have an impact on their children in terms of their behaviours, emotions and other aspects, and are prone to prompting their children to develop similar patterns. Therefore, the use of rejection parenting style by parents will make their children timid and weak, so that they do not dare to show their emotions and lack the ability to manage their emotions well. In addition to this, the more parents use the rejection parenting style, ignoring their children’s emotional demands and life needs, the children feel unresponsive, will hide their true emotions, and often choose to repress and restrain their emotions when they occur. The long-term use of repression and restraint to deal with their own emotions, negative emotions can not be discharged, will impair their emotional regulation ability, reduce the sense of emotional security.

### Relationship between HCA, EI and CQ

Firstly, according to the results of the study, it can be seen that nursing students’ HCA, EI and CQ are all positively correlated two by two. First of all, nursing students’ HCA is significantly positively correlated with EI. It has been confirmed that when nursing students can accurately perceive each other’s emotional changes and express their emotions through words and expressions, and can reasonably control themselves in different situations, they can pay attention to understanding others and their needs, and build a harmonious patient-nurse relationship [40]. Therefore, by stimulating nursing students’ inner emotions, EI allows them to better associate with and understand the emotions and behaviors of others. This understanding can then be transformed into practical actions, a process through which emotions are converted into humanistic caring competence. Nursing students with high EI have higher ability to assess, regulate and utilize their own and other people’s emotions, have higher interpersonal communication ability and the ability to utilize their emotions to achieve their goals, and are more willing to take the initiative in taking care of their patients to promote HCA to improve [41]. Second, HCA was significantly and positively correlated with CQ. Nursing students with strong HCA, when communicating with patients from different cultural backgrounds, will first understand the cultural differences between themselves and the patients, understand the special needs of the patients, realise the importance of multicultural care at an earlier stage, carry out appropriate supportive communication based on this, provide targeted care for patients from different cultural backgrounds through cultural care, accept and respect each other, and implement a caring approach that meets their cultural needs while implementing high quality humanistic care. They also accept and respect each other, and implement care that is in line with their needs and meets their cultural needs while providing high quality humanistic care. Therefore, CQ is inextricably linked to HCA. Finally, the level of EI of nursing students was positively correlated with the level of CQ. It is similar to the results of Drame et al. [28], they can be more sensitive to changes in the emotions of others, establish a good communication atmosphere [42], enhance their own verbal and non-verbal expression skills, continue to learn about the differences between cultures, improve their knowledge of unfamiliar cultures, and have a higher level of CQ.

### The mediating role of HCA

The results of this study showed that the parental rejection dimension was a significant negatively associated with HCA, and that HCA was a significant positively associated with of CQ. The parental emotional warmth dimension was a significant positively associated with HCA, and HCA was a significant positively associated with CQ. First of all, parents’ strict way of disciplining their children or excessive interference in their daily behaviour will cause their children to have a pessimistic and introverted personality, which may contribute to adopt negative behaviours of avoiding interactions with the crowd and lack the courage and awareness of taking the initiative to socialize with others. Nursing students grow up in such a repressive environment, which may contribute to their withdrawn and fragile character, and after entering the clinical internship, their adaptability to the hospital environment will be reduced, which may contribute to them less enthusiastic about their work, and they will refuse to socialise with the patients or other colleagues, resulting in the lack of caring ability, which is directly associated with the quality of their nursing services, and associated with the recovery process of the patients. Whereas, it has been confirmed in Guo Yang et al.’s study [27] that nursing students with high humanistic caring competence are more likely to adapt to the clinical environment, are good at socialising with others, are more inclusive, accept more diverse cultures, provide appropriate care, and improve the degree of intimacy between nurses and patients.

Secondly, this study has confirmed that nursing students’ HCA mediates the relationship between parental rejection of parenting styles and CQ. Therefore, when parents give their children harsh and negative parenting styles, their children feel more negative feelings such as repression, pain, and lack of understanding, lack of active caring for other people, are unwilling to contact other people, and are resistant to socialising with different groups of people, which may contribute to a lack of cultural acceptance and hinders the development of their CQ. On the other hand, parental emotional warmth is a positive way of upbringing, which is mainly manifested in their gentle, tolerant, understanding and respect for their children’s daily behaviours. Therefore, a positive family atmosphere is conducive to the holistic development of nursing students. It fosters character traits such as understanding, gentleness, and consideration, while also may contribute to their social interaction skills. Such an environment encourages them to actively care for others, communicate effectively with diverse groups, and remain open to new experiences. Additionally, it is conducive to their patience and receptiveness in caregiving contexts. These qualities collectively enable nursing students to proactively adapt to different environments, thereby promoting the development of their CQ.

### The mediating role of EI

The results of this study showed that the parental rejection dimension was a significant negative associated with EI. EI was a significant associated with of CQ. The significant chain mediation effect identified in our study provides empirical support for the theoretical integration of Bronfenbrenner’s microsystem theory and the “parent-child transfer” mechanism. It demonstrates that the family environment (microsystem), operationalized through parenting styles, is not only directly associated with CQ but also operates by systematically shaping the child’s internal psychological resources-namely, humanisticompetence (an outward-oriented practical ability) and EI (an inward-oriented regulatory ability) [43]. These resources are subsequently “transferred” and transformed into an individual’s adaptive capabilities within multicultural contexts. This pathway manifests specifically as follows: when parents adopt negative parenting styles such as rejection and denial, their children tend to develop expressive suppression strategies (e.g., avoidance and repression), which hinder their emotional development [43]. This not only directly depletes their cognitive and emotional resources but also weakens their capacity to attend to, understand, and care for others. This scarcity of internal resources, in turn, limits their ability to actively learn, communicate effectively, and recognize cultural nuances in multicultural settings such as clinical internships, ultimately constraining the development of their CQ. Conversely, warm and encouraging parenting styles provide children with an emotionally secure base, fostering the development of positive emotion regulation strategies [44]. This enables them to recover more swiftly from negative emotions, thereby freeing up greater psychological energy to focus deeply on and understand the external world and others. For nursing students, this internal advantage allows them to better cope with the challenges of cross-cultural care, proactively absorb multicultural knowledge, and develop empathetic communication, thereby significantly enhancing their CQ. In summary, the chain mediation model of this study clearly delineates a complete transfer pathway originating from the family microsystem, which shapes core interpersonal and emotional competencies, and ultimately drives the development of cross-cultural adaptive capabilities.

### Implications for nursing education and practice

Based on the findings of this study, the following recommendations are proposed for fostering culturally competent nursing professionals in the future. First, in curriculum development, nursing courses should go beyond just teaching cultural knowledge. They should include theories like developmental ecology. For example, a module called “The Self in Care” can help students reflect on how their upbringing (microsystem) shaped their caring tendencies, emotional reactions, and cultural biases. This makes learning about EI and humanistic care more personal and theory-based. Second, provide targeted skills training. For students who lacked early family support, create specific programs. These can include emotion regulation workshops and structured communication simulations. This helps strengthen their EI and humanistic care skills. Third, improve student support systems. Using a holistic approach in assessments. Pay attention to students’ strengths and challenges in relationships and emotions. Connect them with resources like mentors, counseling, and skill groups. This builds a multi-level support network. Finally, encourage reflective practice throughout the training. Use tools like structured journals or clinical debriefings. Guide students to consciously apply and improve their EI and HCA.

### Limitations

This study has several limitations. Firstly, the sample was drawn exclusively from two provinces in Northwest China. While this provides valuable insights into this specific population, the sociocultural and educational environments may differ from those in other regions of China and internationally, which may restrict the generalizability of the findings. Additionally, as a cross-sectional survey, the research successfully established a chain mediation model, yet the results remain speculative and do not support causal inferences. Furthermore, this study focused on parenting styles as antecedents but did not assess other potential determinants of EI and HCA, such as peer relationships, specific educational interventions, or individual personality traits. Their omission represents another constraint that may affect the comprehensiveness of the model. Therefore, caution is advised when generalizing these results to all undergraduate nursing students. Future research should incorporate replication studies across diverse geographical and cultural settings, along with longitudinal designs and the inclusion of additional contextual and individual-level variables, to further establish the external validity and refine the proposed model.

## Conclusion

Parental rejection is negatively associated with CQ. Parental warmth is positively associated with CQ. HCA and EI significantly mediated the relationship between the parental rejection dimension and CQ. The Parental emotional warmth dimension is positively associated with CQ of undergraduate nursing students. HCA and EI significantly mediated the relationship between the parental emotional warmth dimension and CQ. Therefore, nursing educators should pay attention to the influence of nursing students’family education style on their caring ability and emotional regulation, and adopt appropriate teaching strategies to improve the level of nursing students’ EI and caring competence, so as to improve their CQ.

## Data Availability

The datasets used and/or analysed during the current study are available from the firstand corresponding author on reasonable request.
